# Supplementary material for: Disentangling the complex gene interaction networks between rice and the blast fungus identifies a new pathogen effector
Source: PLoS Biol. 2023 Jan 19;21(1):e3001945. doi: 10.1371/journal.pbio.3001945 (PMC9851567; doi:10.1371/journal.pbio.3001945)

# Fig 7B

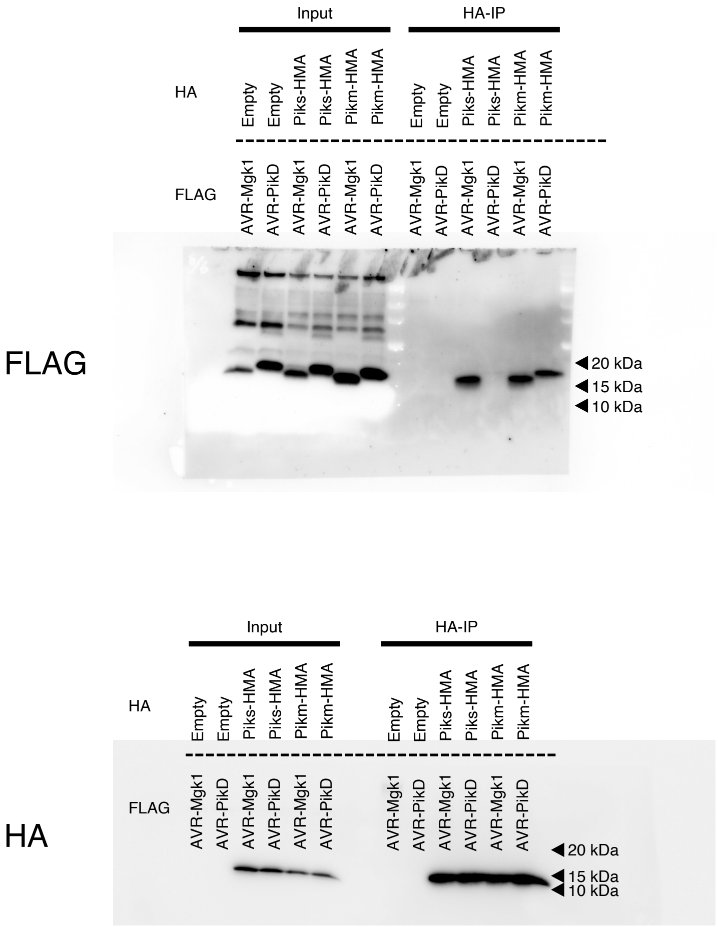
Input
HA-IP
HA
Empty
Empty
Piks-HMA
Piks-HMA
Pikm-HMA
Pikm-HMA
Empty
Empty
Piks-HMA
Piks-HMA
Pikm-HMA
Pikm-HMA
FLAG
AVR-Mgk1
AVR-PikD
AVR-Mgk1
AVR-PikD
AVR-Mgk1
AVR-PikD
AVR-Mgk1
AVR-PikD
AVR-Mgk1
AVR-PikD
AVR-Mgk1
AVR-PikD
FLAG
20 kDa
15 kDa
10 kDa
Input
HA-IP
HA
Empty
Empty
Piks-HMA
Piks-HMA
Pikm-HMA
Pikm-HMA
Empty
Empty
Piks-HMA
Piks-HMA
Pikm-HMA
Pikm-HMA
FLAG
AVR-Mgk1
AVR-PikD
AVR-Mgk1
AVR-PikD
AVR-Mgk1
AVR-PikD
AVR-Mgk1
AVR-PikD
AVR-Mgk1
AVR-PikD
AVR-Mgk1
AVR-PikD
HA
20 kDa
15 kDa
10 kDa

# S8 Fig

Rice *Actin*

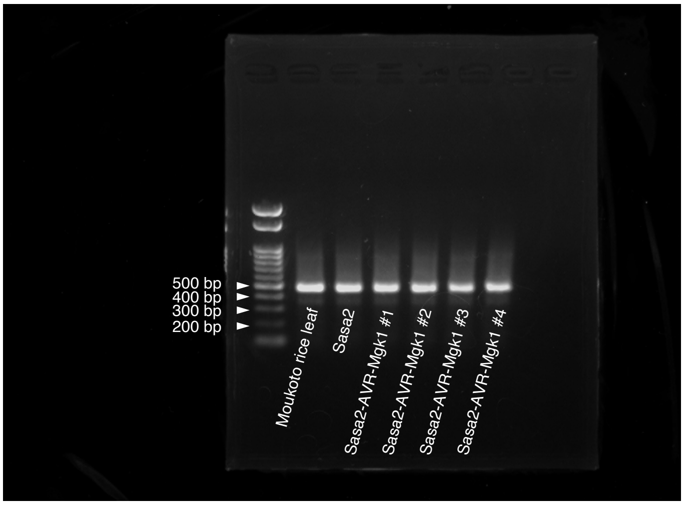


*M. oryzae Actin*

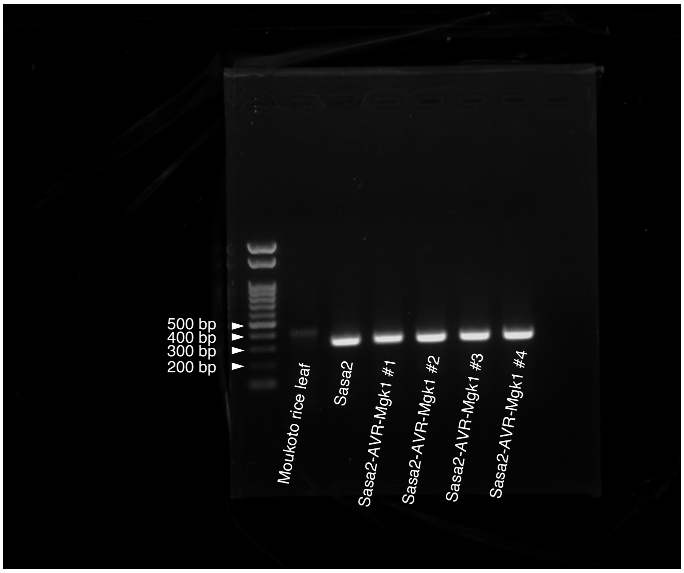


*AVR-Mgk1*

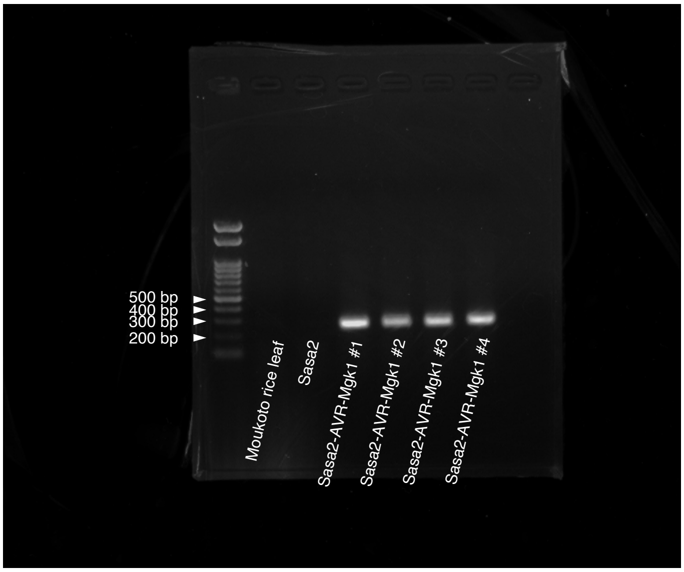

# S13 Fig

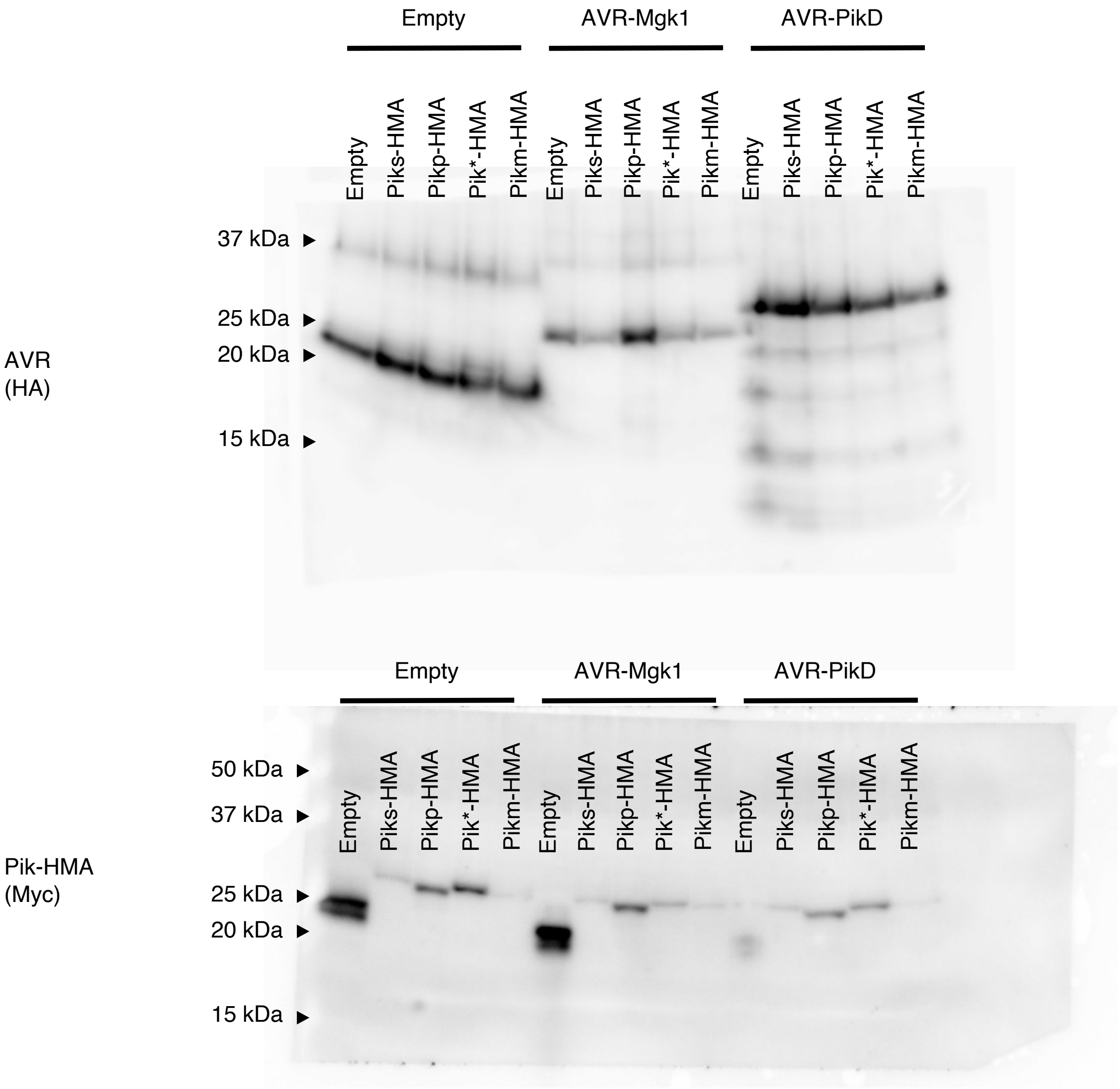
Empty
AVR-Mgk1
AVR-PikD
Empty
Piks-HMA
Pikp-HMA
Pik*-HMA
Pikm-HMA
37 kDa
25 kDa
20 kDa
15 kDa
AVR
(HA)
Empty
AVR-Mgk1
AVR-PikD
50 kDa
37 kDa
25 kDa
20 kDa
15 kDa
Pik-HMA
(Myc)

# S15 Fig

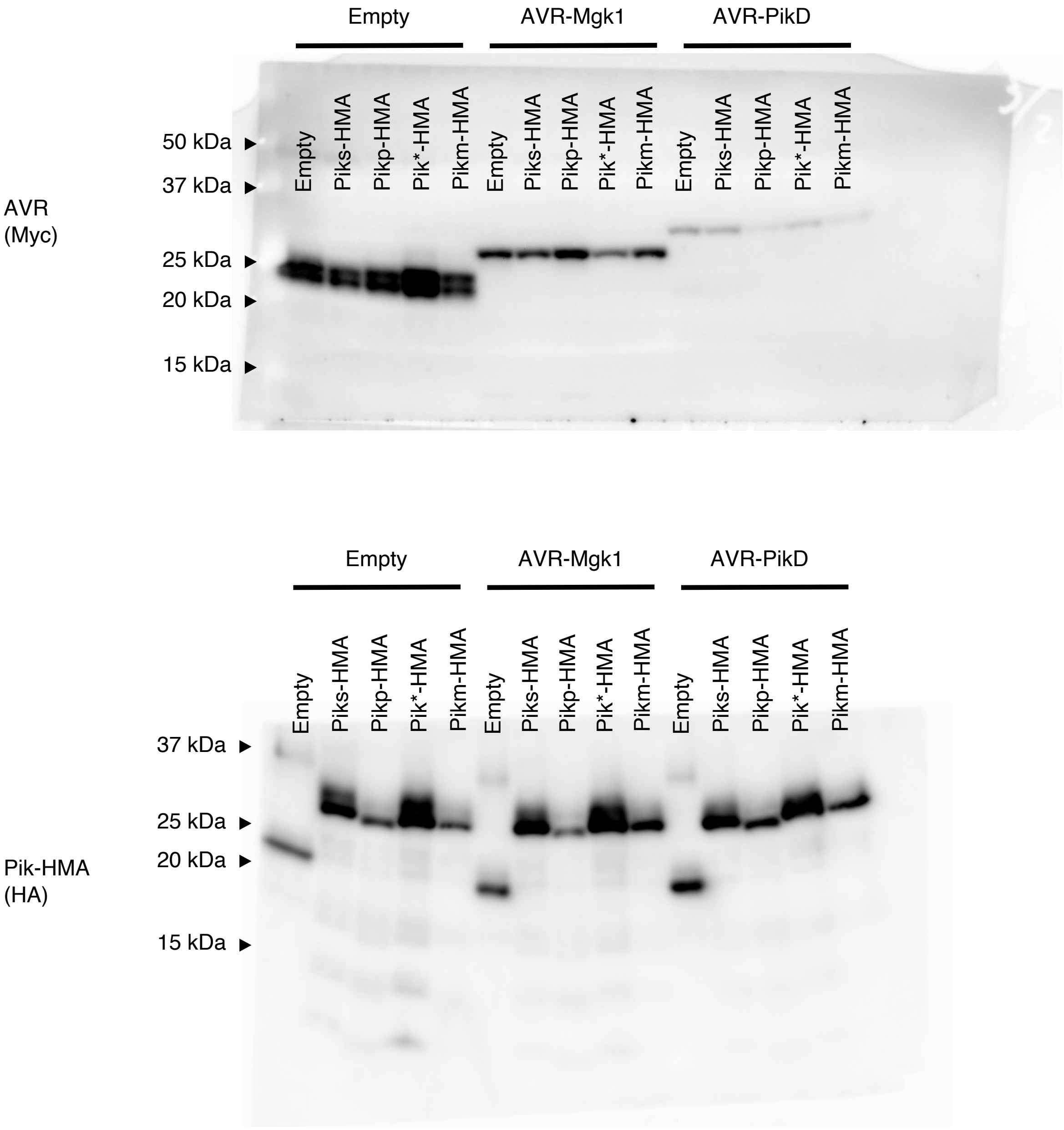

Empty
AVR-Mgk1
AVR-PikD
Empty
Piks-HMA
Pikp-HMA
Pik*-HMA
Pikm-HMA
Empty
Piks-HMA
Pikp-HMA
Pik*-HMA
Pikm-HMA
Empty
Piks-HMA
Pikp-HMA
Pik*-HMA
Pikm-HMA
50 kDa
37 kDa
25 kDa
20 kDa
15 kDa
AVR
(Myc)
Empty
AVR-Mgk1
AVR-PikD
Empty
Piks-HMA
Pikp-HMA
Pik*-HMA
Pikm-HMA
Empty
Piks-HMA
Pikp-HMA
Pik*-HMA
Pikm-HMA
Empty
Piks-HMA
Pikp-HMA
Pik*-HMA
Pikm-HMA
37 kDa
25 kDa
20 kDa
15 kDa
Pik-HMA
(HA)

# S16A Fig

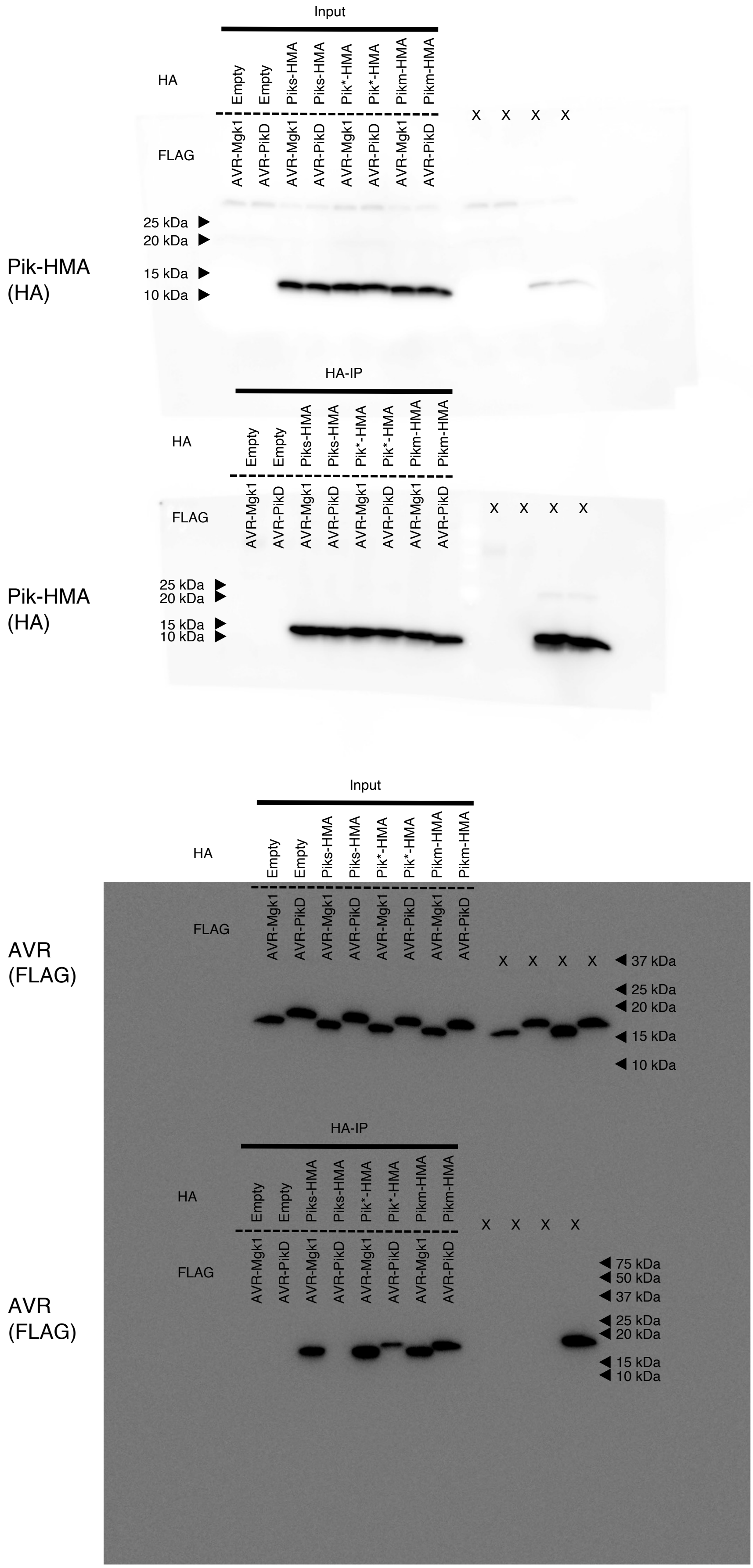
Input
HA
Empty
Empty
Piks-HMA
Piks-HMA
Pik*-HMA
Pik*-HMA
Pikm-HMA
Pikm-HMA
X X X X
FLAG
AVR-Mgk1
AVR-PikD
AVR-Mgk1
AVR-PikD
AVR-Mgk1
AVR-PikD
AVR-Mgk1
AVR-PikD
25 kDa
20 kDa
15 kDa
10 kDa
Pik-HMA (HA)
HA-IP
HA
Empty
Empty
Piks-HMA
Piks-HMA
Pik*-HMA
Pik*-HMA
Pikm-HMA
Pikm-HMA
FLAG
AVR-Mgk1
AVR-PikD
AVR-Mgk1
AVR-PikD
AVR-Mgk1
AVR-PikD
AVR-Mgk1
AVR-PikD
X X X X
25 kDa
20 kDa
15 kDa
10 kDa
Pik-HMA (HA)
Input
HA
Empty
Empty
Piks-HMA
Piks-HMA
Pik*-HMA
Pik*-HMA
Pikm-HMA
Pikm-HMA
FLAG
AVR-Mgk1
AVR-PikD
AVR-Mgk1
AVR-PikD
AVR-Mgk1
AVR-PikD
AVR-Mgk1
AVR-PikD
X X X X
AVR (FLAG)
37 kDa
25 kDa
20 kDa
15 kDa
10 kDa
HA-IP
HA
Empty
Empty
Piks-HMA
Piks-HMA
Pik*-HMA
Pik*-HMA
Pikm-HMA
Pikm-HMA
X X X X
FLAG
AVR-Mgk1
AVR-PikD
AVR-Mgk1
AVR-PikD
AVR-Mgk1
AVR-PikD
AVR-Mgk1
AVR-PikD
AVR (FLAG)
75 kDa
50 kDa
37 kDa
25 kDa
20 kDa
15 kDa
10 kDa

# S16B Fig

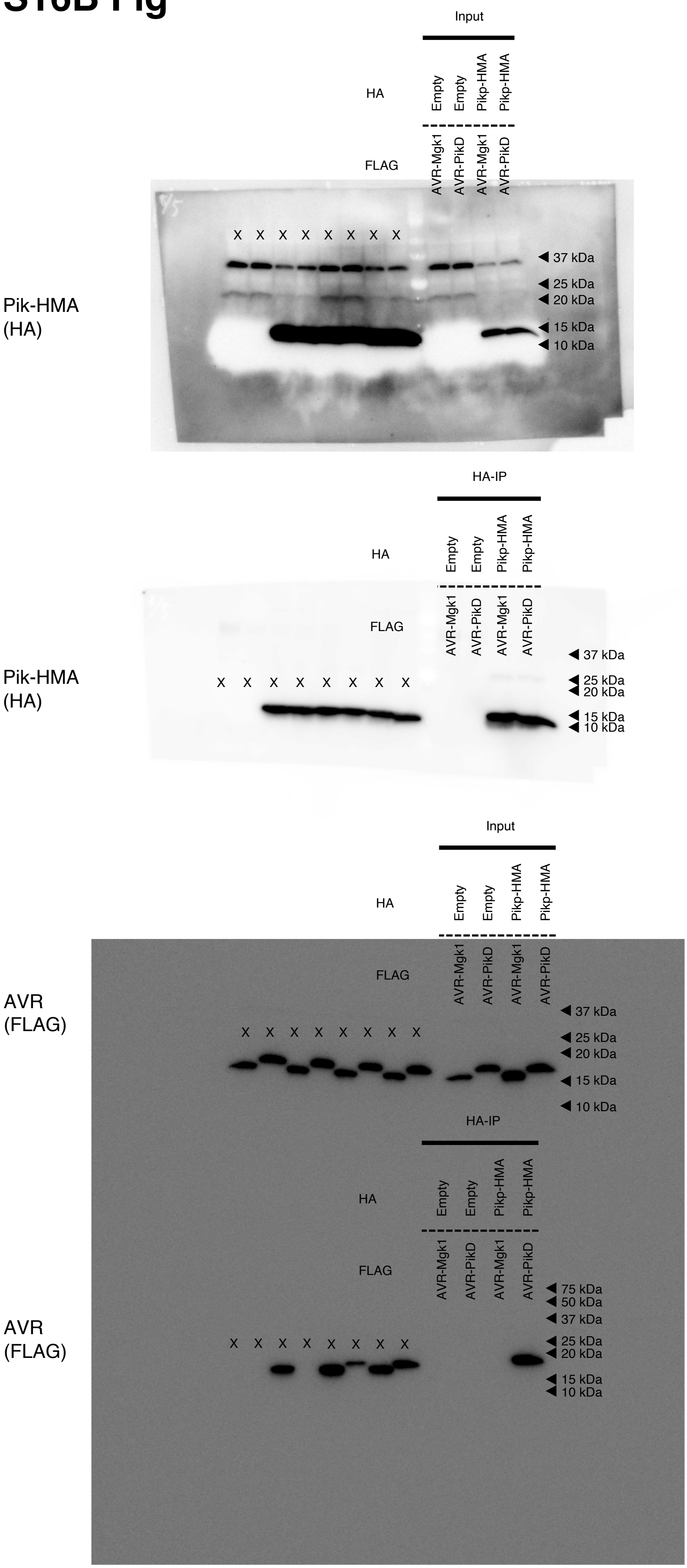
Input
HA
Empty
Empty
Pikp-HMA
Pikp-HMA
FLAG
AVR-Mgk1
AVR-PikD
AVR-Mgk1
AVR-PikD
Pik-HMA (HA)
X X X X X X X X
37 kDa
25 kDa
20 kDa
15 kDa
10 kDa
HA-IP
HA
Empty
Empty
Pikp-HMA
Pikp-HMA
FLAG
AVR-Mgk1
AVR-PikD
AVR-Mgk1
AVR-PikD
Pik-HMA (HA)
X X X X X X X X
37 kDa
25 kDa
20 kDa
15 kDa
10 kDa
Input
HA
Empty
Empty
Pikp-HMA
Pikp-HMA
FLAG
AVR-Mgk1
AVR-PikD
AVR-Mgk1
AVR-PikD
AVR (FLAG)
X X X X X X X X
37 kDa
25 kDa
20 kDa
15 kDa
10 kDa
HA-IP
HA
Empty
Empty
Pikp-HMA
Pikp-HMA
FLAG
AVR-Mgk1
AVR-PikD
AVR-Mgk1
AVR-PikD
AVR (FLAG)
X X X X X X X X
75 kDa
50 kDa
37 kDa
25 kDa
20 kDa
15 kDa
10 kDa

# S16C Fig

## Pik-HMA (HA)

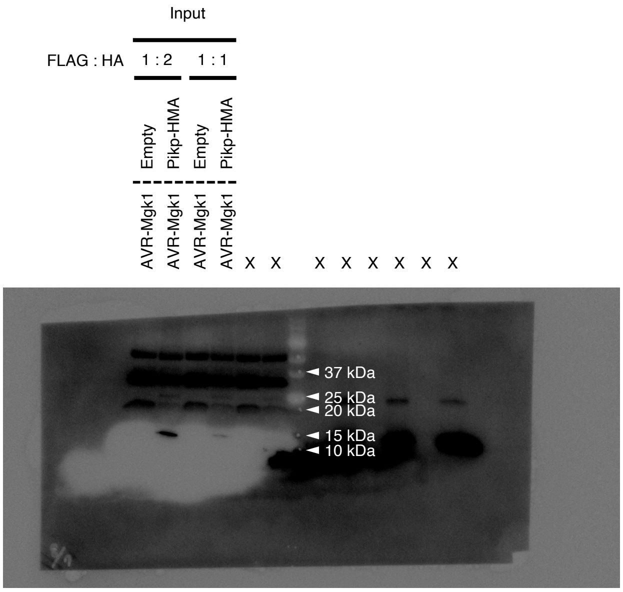


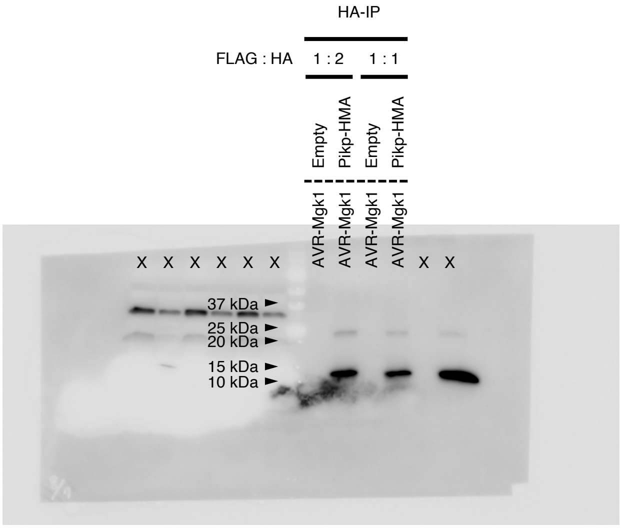


## AVR (FLAG)

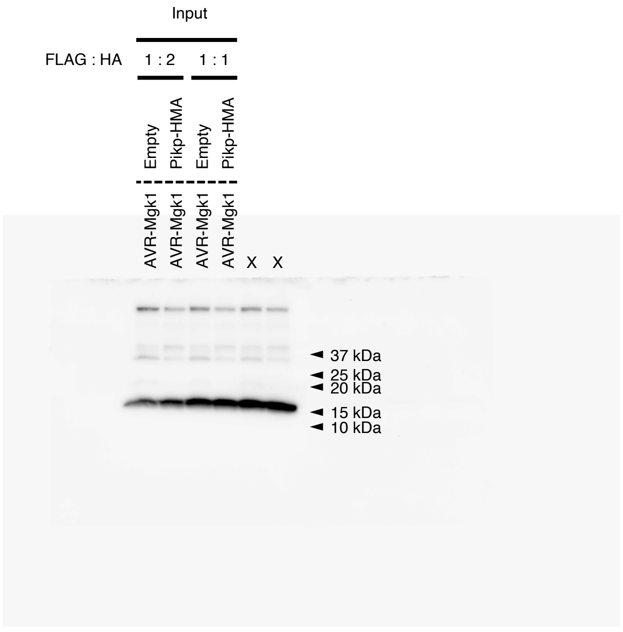


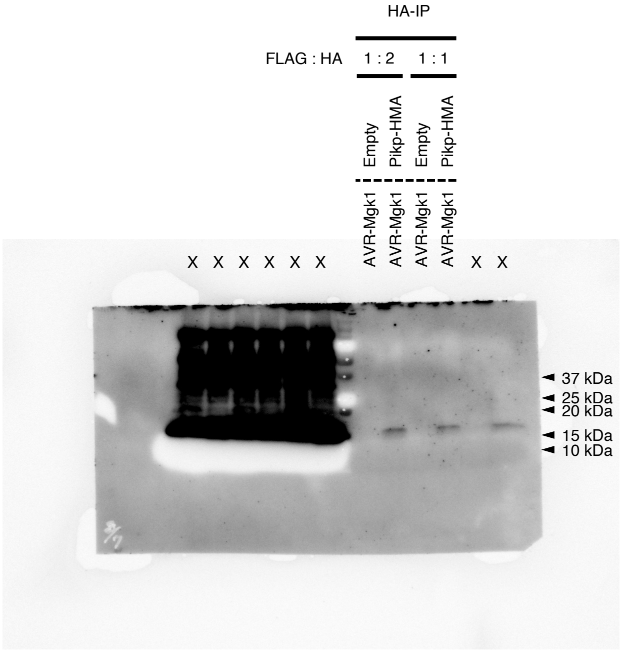

# S18 Fig

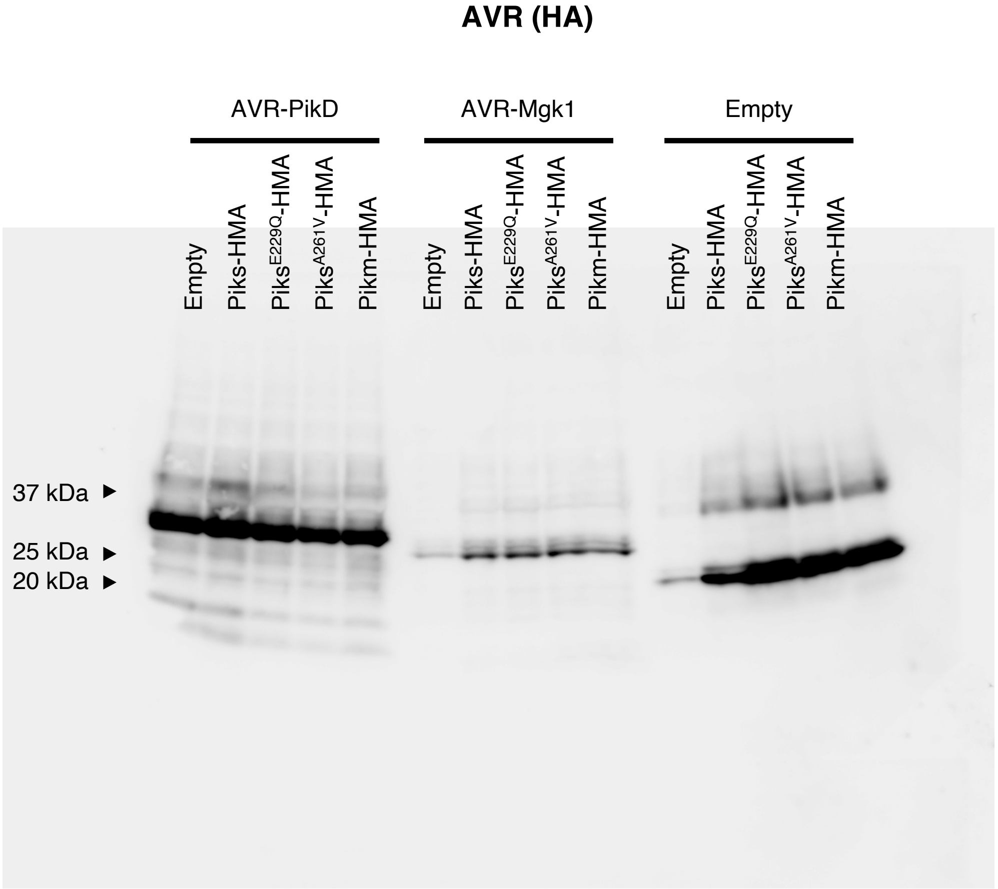
AVR (HA)
AVR-PikD
AVR-Mgk1
Empty
Empty
Piks-HMA
Piks$^{E229Q}$-HMA
Piks$^{A261V}$-HMA
Pikm-HMA
37 kDa
25 kDa
20 kDa

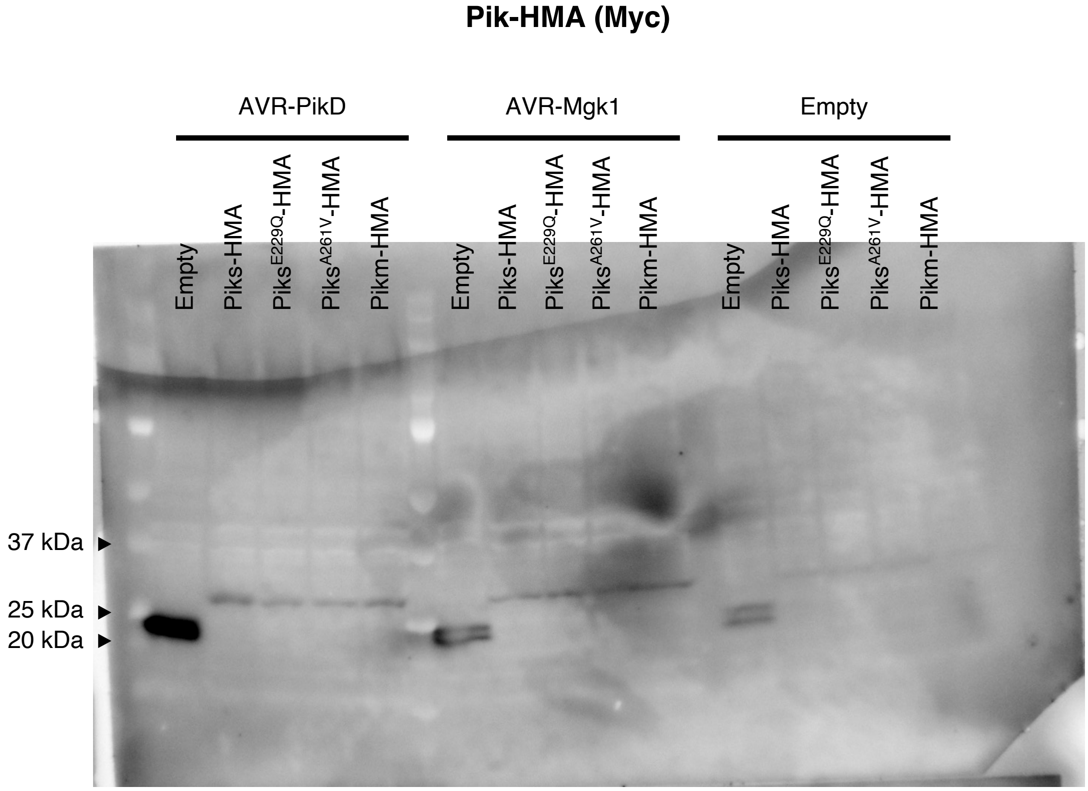
Pik-HMA (Myc)
AVR-PikD
AVR-Mgk1
Empty
Empty
Piks-HMA
Piks$^{E229Q}$-HMA
Piks$^{A261V}$-HMA
Pikm-HMA
37 kDa
25 kDa
20 kDa

# S19 Fig

**AVR (Myc)**

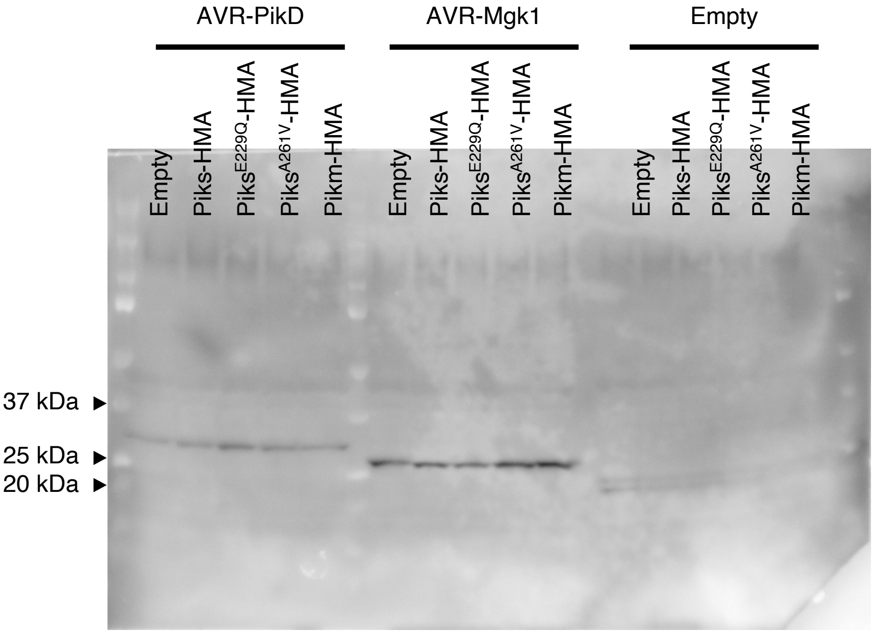


**Pik-HMA (HA)**

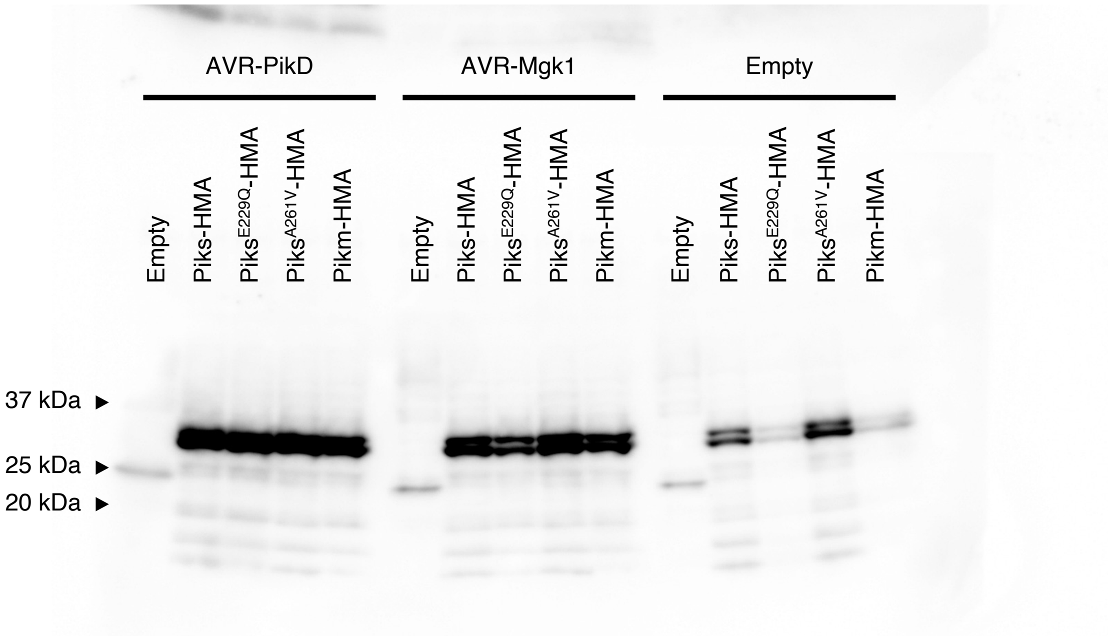

Supplement: S1 Raw images — (PDF) [file pbio.3001945.s030.pdf]
